# Development and clinimetric assessment of a nurse-administered screening tool for movement disorders in psychosis

**DOI:** 10.1192/bjo.2018.55

**Published:** 2018-09-27

**Authors:** Bettina Balint, Helen Killaspy, Louise Marston, Thomas Barnes, Anna Latorre, Eileen Joyce, Caroline S. Clarke, Rosa De Micco, Mark J. Edwards, Roberto Erro, Thomas Foltynie, Rachael M. Hunter, Fiona Nolan, Anette Schrag, Nick Freemantle, Yvonne Foreshaw, Nicholas Green, Kailash P. Bhatia, Davide Martino

**Affiliations:** Neurologist, Sobell Department of Motor Neuroscience and Movement Disorders, UCL Institute of Neurology, UK and Department of Neurology, University of Heidelberg, Germany; Psychiatrist, Division of Psychiatry, Department of Primary Care and Population Health, University College London and Priment Clinical Trials Unit, University College London, UK; Research Associate, Department of Primary Care and Population Health, University College London and Priment Clinical Trials Unit, University College London, UK; Psychiatrist, Department of Psychiatry, Imperial College London, UK; Neurologist, Sobell Department of Motor Neuroscience and Movement Disorders, UCL Institute of Neurology, University College London, UK and Department of Neurology and Psychiatry, Sapienza, University of Rome, Italy; Psychiatrist, Sobell Department of Motor Neuroscience and Movement Disorders, UCL Institute of Neurology, University College London, UK; Research Associate, Department of Primary Care and Population Health and Priment Clinical Trials Unit, University College London, UK; Neurologist, Department of Medical, Surgical, Neurological, Metabolic and Aging Sciences and MRI Research Center SUN-FISM, University of Campania ‘Luigi Vanvitelli’, Italy; Neurologist, Institute of Cardiovascular and Cell Sciences, St George's University, UK; Neurologist, Neurodegenerative Diseases Center (CEMAND) Department of Medicine, Surgery and Dentistry, University of Salerno, Italy; Neurologist, Sobell Department of Motor Neuroscience and Movement Disorders, UCL Institute of Neurology, University College London, UK; Research Associate, Department of Primary Care and Population Health, and Priment Clinical Trials Unit, University College London, UK; Research Nurse, School of Health and Social Care, University of Essex, UK; Neurologist, Department of Clinical Neurosciences, Royal Free Campus, UCL Institute of Neurology, University College London, UK; Statistician, Department of Primary Care and Population Health, University College London and Priment Clinical Trials Unit, University College London, UK; Research Nurse, Camden and Islington NHS Foundation Trust, St Pancras Hospital, UK; Research Nurse, Camden and Islington NHS Foundation Trust, St Pancras Hospital, UK; Neurologist, Sobell Department of Motor Neuroscience and Movement Disorders, UCL Institute of Neurology, University College London, UK; Neurologist, Department of Clinical Neurosciences, University of Calgary, Canada

**Keywords:** Drug interactions and side effects, antipsychotics, clinical neurology

## Abstract

**Background:**

Movement disorders associated with exposure to antipsychotic drugs are common and stigmatising but underdiagnosed.

**Aims:**

To develop and evaluate a new clinical procedure, the ScanMove instrument, for the screening of antipsychotic-associated movement disorders for use by mental health nurses.

**Method:**

Item selection and content validity assessment for the ScanMove instrument were conducted by a panel of neurologists, psychiatrists and a mental health nurse, who operationalised a 31-item screening procedure. Interrater reliability was measured on ratings for 30 patients with psychosis from ten mental health nurses evaluating video recordings of the procedure. Criterion and concurrent validity were tested comparing the ScanMove instrument-based rating of 13 mental health nurses for 635 community patients from mental health services with diagnostic judgement of a movement disorder neurologist based on the ScanMove instrument and a reference procedure comprising a selection of commonly used rating scales.

**Results:**

Interreliability analysis showed no systematic difference between raters in their prediction of any antipsychotic-associated movement disorders category. On criterion validity testing, the ScanMove instrument showed good sensitivity for parkinsonism (90%) and hyperkinesia (89%), but not for akathisia (38%), whereas specificity was low for parkinsonism and hyperkinesia, and moderate for akathisia.

**Conclusions:**

The ScanMove instrument demonstrated good feasibility and interrater reliability, and acceptable sensitivity as a mental health nurse-administered screening tool for parkinsonism and hyperkinesia.

**Declaration of interest:**

None.

## Background

Long-term treatment with antipsychotic medication of patients with an established psychotic illness can cause a range of hypokinetic and hyperkinetic movement disorders. Parkinsonism and akathisia may occur shortly after the beginning of antipsychotic exposure, and may last indefinitely if the exposure continues. Delayed-onset (or tardive) movement disorders associated with antipsychotics comprise a spectrum of abnormal movements cumulatively labelled as tardive dyskinesia, and tardive akathisia.^1^^,^^2^ These usually appear after many months or years of drug treatment, and often do not abate completely, or may even worsen, after treatment withdrawal.^1^^,^^2^ Antipsychotic-associated movement disorders may cause social stigma and have an impact on quality of life.^3^^–^^11^

The prevalence of tardive dyskinesia from trials and naturalistic studies ranges between 13.1% for second-generation antipsychotics and 32.4% for first-generation antipsychotics.^12^^–^^19^ The prevalence of other movement disorders across reports ranges between 23 and 65% for parkinsonism, and between 15 and 30% for akathisia.^17^^,^^18^^,^^20^ The lower prevalence of movement disorders reported with some of the newer antipsychotics has probably contributed to diminished awareness among health professionals.

## Rationale

Movement disorders in established psychosis are still underrecognised. Within a quality improvement programme, a national audit of specialist mental health provider organisations in the UK in 2008 reported that, despite existing national clinical guidelines, 69% of 5804 patients receiving depot/long-acting antipsychotic preparations were not assessed at all for movement disorders in the previous year, and only 4% had been formally evaluated for these manifestations.^21^ This performance improved only in part following educational interventions, suggesting that other factors, besides limited awareness, play a role in shaping health professionals' attitudes towards movement disorders monitoring. In particular, a sufficiently brief and reliable instrument for their systematic screening is lacking. The most popular instruments available in routine clinical practice are validated multiple-item severity rating scales.^22^^–^^25^ Although their use has been adapted for screening purposes, these may be considered too long to administer together.^26^

Although their role within primary and secondary mental health services is still debated,^27^^,^^28^ registered mental health nurses provide a crucial contribution to long-term care, including the provision of psychosocial interventions and health promotion for patients in both in-patient and out-patient settings.^29^ This specific activity has been underexplored in mental health nurses, although their involvement in side-effect screening for long-term antipsychotics could represent a cost-effective strategy.

In this study, we present the development and initial clinimetric evaluation of a new clinical procedure, the ScanMove instrument, for the screening of antipsychotic-associated movement disorders performed by mental health nurses for patients with established psychosis from community services.

## Method

### Development of the ScanMove instrument

The ScanMove instrument was developed by a panel of four neurologists, four psychiatrists and one mental health nurse with expertise in movement disorders associated with antipsychotics. The panel formulated an initial list of diagnostically relevant clinical features of parkinsonism, hyperkinesia (encompassing all types of involuntary movements) and akathisia, based on clinical experience and critical review of existing rating scales. Panellists judged each feature as ‘essential’ or ‘not essential’ for the diagnosis of movement disorder, based on the following questions: ‘does this feature help substantially in the diagnosis?’, ‘is the assessment of this feature sufficiently reliable, feasible and effective to be applied on large clinical scale?’. The content validity of each feature was measured calculating the content validity ratio (CVR) as follows: CVR = (*n*_e_ – *N*/2)/(*N*/2), where *n*_e_ is the number of raters judging the feature as ‘essential’, and *N* is the total number of raters. All features with CVR >0.75 passed content validity assessment at the first round and were included in the instrument. A second round of discussion focused on features with a CVR between 0.5 and 0.75, leading by consensus to a final decision of inclusion/exclusion.

The ScanMove instrument was then operationalised defining type and sequence of the clinical manoeuvres required to assess the selected features, structuring a procedure that could be administered within 15 min. The assessment of each clinical feature led to one of three possible judgements: ‘yes’, ‘no’, ‘unsure’.

### Training of raters

Thirteen registered mental health nurses experienced in mental illnesses in in-patient or community services were trained in the ScanMove instrument through three half-day interactive sessions run by two movement disorder neurologists (D.M. and K.P.B.). The first session provided an overview of the phenomenology of antipsychotic-associated movement disorder using historical patient video recordings. In the other two small group sessions, trainers and trainees reviewed video recordings of the instrument being administered to 20 community psychiatric patients.

### Reliability assessment

Thirty adult patients with consenting capacity from community services within three National Health Service mental health trusts in North and West London were recruited for interrater reliability testing, enrolling eligible patients consecutively. Inclusion criteria were: (a) one of the following DSM-V diagnoses:^30^ schizophrenia, schizophreniform disorder, schizoaffective disorder, or delusional disorder; (b) documented exposure for >3 months to ≥1 antipsychotic drug; (c) having an allocated care coordinator within a community rehabilitation team or residential service; (d) absence of neurological diagnoses causing movement disorder. All patients were administered the ScanMove instrument by the evaluating neurologist (B.B.). The assessment was recorded using the same video camera and audiovisual settings. Ten trained mental health nurses rated the video recordings completing the ScanMove instrument summary sheet. Ratings provided an aggregated score (1 point per item) and a dichotomous judgement (≥1 item, presence) separately for parkinsonism, hyperkinesia and akathisia.

### Criterion and concurrent validity assessment

Patients from the same community services were selected with the same criteria, and underwent a single study visit. Sociodemographic data, psychiatric diagnoses and information on medication exposure during the previous year were collected for each participant by one of the trained mental health nurses. Subsequently, the same nurse administered the ScanMove instrument. After a brief intermission, the evaluating movement disorder neurologist used the same clinical manoeuvres applied during ScanMove instrument administration as well as reference validated rating scales. These scales were selected by panellists based on their frequency of routine application in psychiatric practice, and included the Modified Simpson Angus Scale (MSAS) for extrapyramidal side effects/parkinsonism,^23^ the Abnormal Involuntary Movements Scale (AIMS) for dyskinesia and adventitious movements,^22^ and the Barnes Akathisia Rating Scale (BARS) for akathisia.^24^

The MSAS is a ten-item scale in which each item is scored from 0 to 4; the total score is obtained dividing by ten the sum of the scores of the ten items, therefore ranging between 0 and 4. A revised version of this scoring was also used for analysis, which omitted items seven and ten, judged by the panel not specifically relevant to parkinsonism. For this revised version the total score was obtained, dividing the sum of the scores of the retained by ten, hence leaving the total score range of 0–4 unchanged. Only the first seven items of the AIMS were used for analysis; these are scored 0,  absent to 4, severe, yielding a total score range of 0–28. The BARS uses three questions with response ratings from 0, absent to 3,  severe; these are summed to give a score ranging between 0 and 9; only the global scale was used in the analysis, dichotomised to those scoring ≥2 (defining ‘clinically relevant’ akathisia) versus those scoring less than 2. The overall duration of scale administration ranged between 10 and 15 min.

Nurses and the evaluating neurologist entered their evaluation on a web-based database, remaining masked to each other's ratings for the study duration. The web-based database, built using Sealed Envelope, included range, logic and consistency checks and, for closed questions, provided a number of fixed options, all of which minimised data entry errors. Data were further checked by the main statistician in the study team (L.M.) who then liaised with the study coordinator (D.M.) to rectify pending issues with impossible values or inconsistent data entered.

### Statistical analyses

Descriptive statistics were calculated for all variables, items within the measures, their total scores and the ScanMove instrument. Any systematic difference between raters on the 30 patients' video recordings was estimated through an interaction test in a model with repeated patient measures. For the same video recordings, the relationship of positive detection between nurses and neurologist was estimated in non-linear models with repeated measures for raters to estimate the diagnostic odds ratio (OR). The diagnostic odds ratio is the ratio of the odds of the test being positive if the participant has a disease relative to the odds of the test being positive if the participant does not have the disease. As this is estimated using mixed models to account for rater, the confidence interval on the diagnostic odds ratio accounts for the between- and within-rater variability.

To test criterion validity of the nurse-based dichotomous judgement on the presence/absence of parkinsonism, hyperkinesia and akathisia derived from the ScanMove instrument (≥1 item, presence), we calculated the area under the curve, along with sensitivity, specificity and percentage correctly identified and their respective 95% confidence intervals, using as gold standard the neurologist's dichotomous judgement based on the ScanMove instrument.

For concurrent validity analysis of the nurses' ScanMove additive score, mixed-effect linear (for MSAS and AIMS as outcome measure) or logistic (for BARS as outcome measure) regression models were used, accounting for differential rating across nurses with a random intercept. For these models, ‘unsure’ ratings in the ScanMove instrument were recoded to ‘no’. Gold-standard scale scores were calculated for the original of each scale, as well as for the revised version of MSAS. The revised version of MSAS was also used to assess first-order interactions between ScanMove items; these were considered using backwards selection, based upon a criterion for model entry of *P*<0.20. There was no interaction analysis for BARS positive scores. Models within each outcome measure were compared using the Akaike information criterion (AIC),^31^ for which the best fitting model is the one with the lowest AIC. Once the best fitting models were established for MSAS and AIMS, the fitted values (fixed effect + contribution for the random effect) were plotted against the actual scores. Finally, Bland–Altman plots were constructed.^32^ For the BARS models, the area under the curve was calculated along with the sensitivity, specificity and percentage correctly identified and their respective 95% confidence intervals. Analyses used Stata version 14.2 or SAS version 9.4. The ScanMove study was approved by the NRES Ethics Committee London – Bromley Authority (authorisation nr. 14/LO/0835).

## Results

### Content validity

The content validity testing led to the selection of 31 clinical features diagnostically relevant for movement disorder screening (11 for parkinsonism, 14 for hyperkinesia, 6 for akathisia). The new screening procedure was subsequently operationalised into a checklist of 38 questions that captured the outcome for each of the 31 features (Table 1).

**Table 1:** Item per item frequency distribution of movement disorders characteristics detected by the nurse-administered ScanMove instrument (*n* = 635)

ScanMove instrument item	*n*	%
1. *When walking* Is the arm swing reduced (even on one side only)?	350	55
2. *When walking* Is the stride length reduced (even on one side only)?	126	20
3. *When walking* Does the patient shuffle his/her feet?	88	14
4. Does the patient walk with a stooped trunk?	112	18
5. *When walking* Is the patient's head tilting back or to one side?	35	6
6. *When walking* Do you notice any abnormal movements of the face (such as grimacing, pursing and smacking of the lips, chewing and lateral movements of the jaw, tongue protrusion)?	82	13
7. *When walking* Do you notice any abnormal movements of the limbs (such as shaking, twitching or twisting of hands or feet)?	111	17
9. *When standing* Does the patient have any purposeless movements of the legs, such as marching or stamping movements, walking on-the-spot, twitchy, jerky movements?	73	11
10. *When standing* Does the patient's body keep rocking side to side?	43	7
11. *When standing* Does the patient keep pacing around the room leaving his/her spot despite the instruction to stand still?	14	2
13. *When standing* Is the patient's head tilting back or to one side?	36	6
14. *When standing* Do you notice any abnormal movements of the face (such as grimacing, pursing and smacking of the lips, chewing and lateral movements of the jaw, tongue protrusion)?	114	18
15. *When standing* Do you notice any abnormal movements of the limbs (such as shaking, twitching or twisting of hands or feet)?	200	31
17. *When sitting* Does the patient have any purposeless movements of the legs, such as shuffling, jiggling, trampling of the legs?	54	9
18. *When sitting* Does the patient get up out of the chair despite the instruction to sit down?	5	1
20. *When sitting* Is the patient's head tilting back or to one side?	42	7
21. *When sitting* Do you notice any abnormal movements of the face (such as grimacing, pursing and smacking of the lips, chewing and lateral movements of the jaw, tongue protrusion)?	142	22
22. *When sitting* Do you notice any abnormal movements of the limbs (such as shaking, twitching or twisting of hands or feet)?	200	31
24. *When sitting* Does the patient's body keep rocking side to side?	15	2
25. Do the patient's finger tapping movements become smaller as he/she carries on with the task?	338	53
26. If yes, does the patient's finger tapping become also slower as he/she carries on with the task?	243	38
27. Do the patient's foot tapping movements become smaller as he/she carries on with the task?	181	29
28. If yes, does the patient's foot tapping become also slower as he/she carries on with the task?	144	23
29. *While keeping mouth open* Do you notice any abnormal movements in the face (such as grimacing, pursing and smacking of the lips, chewing and lateral movements of the jaw, tongue protrusion)?	143	23
31. *While keeping mouth open* Do you notice any excessive pooling of saliva in the mouth, or is there any drooling of saliva outside of his/her mouth?	22	3
32. Is his/her voice excessively soft?	31	5
33. With the patient relaxed and not actively contracting his/her muscles, do you feel any resistance while doing these manoeuvres?	141	22
34. *While holding arms outstretched or in front of chest with each elbow out to the side* Is the patient's head tilting back or to one side?	28	4
35. *While holding arms outstretched or in front of chest with each elbow out to the side* Do you notice any abnormal movements of the face (such as grimacing, pursing and smacking of the lips, chewing and lateral movements of the jaw, tongue protrusion)?	133	21
36. *While holding arms outstretched or in front of chest with each elbow out to the side* Do you notice any abnormal movements of the limbs (such as shaking, twitching or twisting of hands or feet)?	389	61
38. Do you notice any abnormal shaking, twitching, or twisting of the hands while writing or drawing?	195	31

### Reliability assessment

The neurologist's judgement on the 30 video-recorded patients identified parkinsonism in 22, hyperkinesia in 28 and akathisia in 4. There was no systematic difference between the ten nurses in their prediction of any movement disorder category (parkinsonism *P* = 0.65; hyperkinesia and akathisia *P* = 0.99). The diagnostic odds ratios expressing the relationship between nurses' and neurologist's dichotomous judgement on the same 30 video recordings were 6.75 (95% CI 3.3–13.8, *P* = 0.0002) for parkinsonism, 8.60 (95% CI 3.5–21, *P* = 0.0004) for hyperkinesia and 32.7 (95% CI 11.4–94.1, *P* < 0.0001) for akathisia.

### Feasibility

The ScanMove instrument demonstrated good feasibility. Data collection could be completed in 635 of 647 patients recruited. Twelve (1.9%) dropped out during data collection because of insufficient adherence: 5 (0.8%) during the ScanMove procedure and 7 (1.1%) during the neurologist's procedure. The duration of administration ranged between 12 and 17 min, although it was kept below 15 min in 95% of the assessments; the duration of administration did not significantly differ across nurses (data not shown).

### Criterion validity

The majority of the 635 participants were men (70%), with a mean age of 45 years (s.d. = 12; Table 2). Just under half of participants were White (49%) and 30% were Asian. Just over 80% of participants had a primary diagnosis of schizophrenia. The most frequently used antipsychotic was clozapine (45%), followed by risperidone (30%), olanzapine (24%) and aripiprazole (20%); 38% of patients had been exposed to anticholinergic drugs.

**Table 2:** Summary of demographic and clinical characteristics of the clinical sample for the field validation of the ScanMove instrument (*n* = 635)

Variable	
Men, *n* (%)	443 (70)

Ethnicity, *n* (%)	
White	312 (49)
Black	68 (11)
Asian	191 (30)
Other	64 (10)

Highest educational attainment, *n* (%)	
No qualifications	179 (28)
GCSE or equivalent	163 (26)
A Level or equivalent	92 (14)
NVQ or equivalent	53 (8)
HNC/ HND or equivalent	27 (4)
Degree	66 (10)
Higher degree	31 (5)
Other	24 (4)

Years of education, median (IQR)	12 (11–15)

Primary diagnosis, *n* (%)	
Schizophrenia	521 (82)
Schizophreniform disorder	3 (0.5)
Schizoaffective disorder	92 (14)
Delusional disorder	19 (3)

Secondary diagnosis, *n* (%)	173 (28)^a^

Antipsychotic drug, number ever exposed (%)	
Amisulpride	88 (14)
Aripiprazole	130 (20)
Chlorpromazine	28 (4)
Clozapine	285 (45)
Flupentixol	81 (13)
Flupentixol decanoate	7 (1)
Fluphenazine	6 (1)
Fluphenazine decanoate	9 (1)
Haloperidol	103 (16)
Haloperidol decanoate	9 (1)
Levomepromazine	2 (0.3)
Olanzapine	154 (24)
Paliperidone	27 (4)
Pipotiazine palmitate	23 (4)
Prochlorperazine	1 (0.2)
Quetiapine	63 (10)
Risperidone	191 (30)
Sulpiride	33 (5)
Thioridazine	1 (0.2)
Trifluoperazine	3 (0.5)
Zuclopenthixol	100 (16)
Zuclopenthixol decanoate	15 (2)

Anticholinergics	240 (38)

GCSE, General Certificate of Secondary Education (usually achieved at age 16); A level, Advanced level (usually achieved at age 18); NVQ, National Vocation Qualification (usually achieved at age 19); HNC, Higher National Certificate/HND, Higher National Diploma (usually achieved at age 22); IQR, interquartile range.

a. Of the 615 participants with data for secondary diagnosis.

From the nurses' rating using the ScanMove instrument (Table 1), the most common item detected was ‘abnormal limb movements’ (61%), followed by ‘reduced arm swing’ (55%), ‘reduced amplitude’ and ‘reduced speed’ on finger tapping (53%), and ‘reduced speed’ on foot tapping (38%); the least common clinical feature was ‘rising out of a chair despite being asked to sit’ (1%).

Using the most lenient ≥1 item cut-off, a ScanMove instrument-based diagnosis of any of the three movement disorders categories explored was formulated by nurses for 598 patients (94%) and by the neurologist for 585 (92%). In total, 75 (11.8%) and 111 (17.5%) patients were judged to manifest all three categories of movement disorders by nurses and by the neurologist, respectively. A diagnosis of parkinsonism was formulated by the nurse using the ScanMove instrument in 502 (79%) patients. The neurologist identified parkinsonism with the ScanMove instrument in 305 (48%) patients. Compared with the ScanMove neurologist judgement, the ScanMove nurse judgement showed high sensitivity (90.1%), but low specificity (30.7%), and the area under the curve (C statistic) was 0.60 (95% CI 0.57–0.63).

Hyperkinesia was diagnosed in 515 (81%) patients by the nurse using the ScanMove instrument. The neurologist identified hyperkinesia with the ScanMove instrument in 528/635 (83%) patients. The ScanMove nurse judgement showed a sensitivity of 88.8%, but a lower specificity of 58.5%, with an area under the curve of 0.74 (95% CI 0.69–0.79).

Finally, akathisia was diagnosed in 134/635 (21%) patients by the nurse using the ScanMove instrument. The neurologist identified akathisia in 184/635 (29%) patients using the ScanMove instrument, and in 155/635 (24.4%) patients using the cut-off score of 2 on the BARS. The ScanMove nurse judgement showed low sensitivity (38.3%), but greater specificity (86.3%); the area under the curve was 0.62 (95% CI 0.58–0.66).

Applying a more restrictive cut-off of ≥2 items to the diagnosis of parkinsonism and hyperkinesia led to an increase in specificity (from 23.5 to 56.8% for parkinsonism; from 58.5 to 83.4% for hyperkinesia), but with a decrease in sensitivity (from 93.6 to 65.2% for parkinsonism; from 88.8 to 56.5% for hyperkinesia).

### Concurrent validity

From the neurologist's rating (supplementary Table 1, available at https://doi.org/10.1192/bjo.2018.55), the median overall score of the MSAS was 0.20 (interquartile range (IQR) = 0.10–0.40) for the original 10-item version, and 0.13 (IQR=0.00–0.38) for the revised 8-item version. The overall median AIMS score using the first seven items only was 0 (IQR = 0–4). A quarter of participants were BARS (akathisia) positive.

The mixed-effects linear regression model in which the ScanMove score best predicted the revised MSAS score with interactions included all 11 parkinsonism-specific ScanMove items (supplementary Table 2). The ScanMove item that made the greatest contribution to the MSAS in all models without interactions was the muscle tone assessment (item 33). However, when the fitted values were plotted against MSAS scores, no obvious relationship between the actual scores on the revised MSAS and the fitted values from the model was seen. The Bland–Altman plot yielded a mean difference of −1.59 × 10^−9^ (s.d. = 0.26) and 95% limits of agreement of −5 to 5, indicating low agreement between MSAS score and fitted values.

Similar findings were obtained for AIMS score as outcome. The mixed-effects linear regression model in which the ScanMove score best predicts the AIMS score with interactions included all 14 hyperkinesia-specific ScanMove items (supplementary Table 3). When the fitted values from the model were plotted against AIMS score, no obvious relationship was seen. The Bland–Altman plot yielded a mean difference of 5.65 × 10^−9^ (s.d. = 2.7, and 95% limits of agreement of −5 to 5, also indicating low agreement between AIMS score and fitted values.

The mixed-effects logistic regression model in which the ScanMove score best predicted the BARS dichotomous outcome included all six akathisia-specific ScanMove items. Of note, some of these items were reported in a low number of participants (Table 1). The area under the curve for the best fitting model (supplementary Table 4) was 0.72 (95% CI 0.67–0.77). For this model the optimum sensitivity was 63.8% (95% CI 55.6%–71.4%) and specificity 67.8% (95% CI 63.4%–72.1%).

## Discussion

### Main findings

In this study we developed a screening tool (ScanMove instrument) for movement disorder in patients with established psychosis, conceived for use by mental health nurses. Item selection and operationalisation were conducted by a multidisciplinary panel of movement disorder neurologists, psychiatrists with extensive clinical experience of such movement disorders and a mental health nurse. Clinical features judged to be diagnostically relevant for parkinsonism, hyperkinesia and akathisia were assessed across different functional states or body locations, in order to optimise the sensitivity of the instrument.

The ScanMove instrument administered by the movement disorder neurologist identified at least one of the three movement disorder categories in 92% of the 635 screened community patients with psychosis. This frequency was very similar to the one obtained by mental health nurses using the same instrument. Although it is likely that only a subgroup of these patients will require therapeutic intervention for their movement disorder, the frequency estimates obtained using our screening instrument support the need for greater attention regarding movement disorder from mental health professionals, at least in community-dwelling patients with established psychosis.

Interrater reliability analysis did not identify any systematic difference between raters on the scores for each movement disorder category. An important limitation of this analysis is that the direct muscle tone assessment of rigidity could not be performed using video recordings. Throughout field validity testing, the ScanMove instrument showed high feasibility, with a small number of missing values and a narrow range of administration time that was consistent with the developers’ aim.

Our criterion validity analysis showed that the dichotomous diagnostic judgement using the most lenient cut-off (≥1 item for each diagnostic category) was moderately to highly sensitive, but not specific, in diagnosing parkinsonism and hyperkinesia, when compared with the neurologist's dichotomous judgement. When a more restrictive cut-off of ≥2 items was used to define positive detection of parkinsonism or hyperkinesia, the ScanMove instrument improved in specificity, but at the cost of lower sensitivity, diminishing its value as a screening instrument. Based on this sensitivity analysis, the nurse-administered ScanMove instrument appears to be sufficiently accurate in ruling out parkinsonism and hyperkinesia in this patient population. However, the low specificity values indicate that the diagnoses of parkinsonism and hyperkinesia obtained using the nurse-administered ScanMove instrument should always be confirmed by a physician.

Different considerations should be made with respect to akathisia, for which the diagnostic accuracy of the nurse-administered ScanMove instrument was less satisfactory at the ≥1 item cut-off, suggesting limitations in the content of the items specifically related to akathisia and/or greater training requirements to optimise rating proficiency of akathisia among nurses.

For concurrent validity testing, we evaluated how the ScanMove instrument predicts the outcome of a comprehensive reference procedure yielding a severity score for parkinsonism and hyperkinesia and a binary outcome for akathisia. The composition of this reference procedure aimed to reproduce, to the best of our abilities, the standard practice of psychiatrists working in the UK National Health Service. Importantly, the AIMS evaluates all hyperkinesia with the exception of tremor, which was detected in 47% of patients by item 8 of the MSAS, and contributed substantially to the 83% frequency of hyperkinesia detected by the neurologist's dichotomous judgement. Our results suggested that the ScanMove instrument does not yield quantitative scores that are useful to predict the scores on our reference instruments. With respect to parkinsonism and hyperkinesia, this finding can partly be explained by important differences in their content between the ScanMove instrument and the MSAS and AIMS. The assessment of parkinsonism using MSAS is skewed towards rigidity and tremor, without taking bradykinesia into account, a core feature of parkinsonism included in the ScanMove instrument. In addition, tremor is included in the hyperkinesia subscore whereas the AIMS specifically excludes tremor from hyperkinesia rating. Not surprisingly, the ScanMove item that contributed most to the prediction of the MSAS score was the one examining rigidity. Therefore, lack of agreement between the two scales is likely to be at least based on differences in content and grouping of questions.

### Cost-effectiveness

When delivered by mental health nurses, the ScanMove instrument could provide the capability to increase the proportion of patients assessed for movement disorder with a minimal increase in costs to the services. Assuming that screening is conducted by a mental health nurse, the cost for the 15 min of patient contact required to conduct the screen is £9.25 in 2016 GBP.^33^ Across 1000 patients and using the prevalence, sensitivity and specificity for hyperkinesia, for example, the total cost of a mental health nurse using ScanMove would be £9250. Based on observations from our sample, 808 patients of the 1000 would be identified as potentially having hyperkinesia and referred to the consultant psychiatrist for further assessment (5 min review of notes and 15 min for ScanMove), for a total cost of £29 073 for the consultant psychiatrist assessment, and a cost of £38 323 in total. If current practice of the 30 min assessment by a consultant psychiatrist at a cost of £54 was to be conducted for the same 1000 patients, the total cost would be £54 000. As a result, ScanMove presents a feasible and lower cost way to increase yearly screening of patients for movement disorder, plus referral and treatment.

### Implications

In conclusion, the mental health nurse-administered ScanMove instrument demonstrated good feasibility and interrater reliability and acceptable sensitivity as a screening tool for parkinsonism and hyperkinesia in patients with established psychosis. Sensitivity for akathisia was less satisfactory. In routine clinical practice, it may represent a useful aid in the selection of those patients warranting review by a physician for the management of these motor manifestations.

Further work is needed to evaluate whether a more extensive training programme for mental health nurses in the ScanMove instrument might increase its overall specificity, or its sensitivity for the diagnosis of akathisia. With regard the latter, using the tool in combination with the BARS may be an option, although the BARS has not been validated as yet for mental health nurse use. Alternatively, future work could aim at a revised content for the akathisia items to improve this specific aspect of the ScanMove tool.

Cost-effectiveness appears promising, but requires further investigation. In order to support its dissemination and implementation, future research should compare the cost-effectiveness and the impact on management decision-making and quality of life of use of the ScanMove instrument compared with routine standards of care.

## Supplementary material

For supplementary material accompanying this paper visit http://dx.doi.org/10.1192/bjo.2018.55. click here to view supplementary material
