# Supplementary material for: Development and clinimetric assessment of a nurse-administered screening tool for movement disorders in psychosis
Source: BJPsych Open. 2018 Sep 27;4(5):404–10. doi: 10.1192/bjo.2018.55 (PMC6171333; doi:10.1192/bjo.2018.55)
Supplement: Supplementary file 1 [file S2056472418000558sup001.docx]

**Supplementary Table 1.** Item per item score distribution on the Modified Simpson Angus Scale (MSAS), Abnormal Involuntary Movements Scale (AIMS), and Barnes Akathisia Rating Scale (BARS). IQR: interquartile range.

| ***MSAS*** |  |  |
| --- | --- | --- |
| *1. Gait* |  |  |
| Normal | 384/634 | 61 |
| Diminution in swing while the patient is walking | 214/634 | 34 |
| Marked diminution in swing with obvious rigidity in the arm | 31/634 | 5 |
| Stiff gait with arms held rigidly before the abdomen | 3/634 | 0.5 |
| Stooped shuffling gait with propulsion and retropulsion | 2/634 | 0.3 |
|  |  |  |
| *2. Arm Dropping* |  |  |
| Normal, free fall with loud slap and rebound | 479/630 | 76 |
| Fall slowed slightly with less audible contact and little rebound | 120/630 | 19 |
| Fall slowed, no rebound | 26/630 | 4 |
| Marked slowing, no slap at all | 5/630 | 1 |
| Arms fall as though against resistance; as though through glue | 0/630 | 0 |
|  |  |  |
| *3. Shoulder Shaking* |  |  |
| Normal | 598/634 | 94 |
| Slight stiffness and resistance | 32/634 | 5 |
| Moderate stiffness and resistance | 4/634 | 1 |
| Marked rigidity with difficulty in passive movement | 0/634 | 0 |
| Extreme stiffness and rigidity with almost a frozen shoulder | 0/634 | 0 |
|  |  |  |
| *4. Elbow Rigidity* |  |  |
| Normal | 559/634 | 88 |
| Slight stiffness and resistance | 61/634 | 10 |
| Moderate stiffness and resistance | 11/634 | 2 |
| Marked rigidity with difficulty in passive movement | 2/634 | 0.3 |
| Extreme stiffness and rigidity with almost a frozen elbow | 1/634 | 0.2 |
|  |  |  |
| *5. Wrist Rigidity or Fixation of Position* |  |  |
| Normal | 564/635 | 89 |
| Slight stiffness and resistance | 62/635 | 10 |
| Moderate stiffness and resistance | 6/635 | 1 |
| Marked rigidity with difficulty in passive movement | 2/635 | 0.3 |
| Extreme stiffness and rigidity with almost a frozen wrist | 1/635 | 0.2 |
|  |  |  |
| *6. Head Rotation* |  |  |
| Loose, no resistance | 574/635 | 90 |
| Slight resistance to movement | 44/635 | 7 |
| Resistance is apparent and the time of rotation is shortened | 10/635 | 2 |
| Resistance is obvious and rotation is slowed | 3/635 | 0.5 |
| Head appears stiff and rotation is difficult to carry out | 4/635 | 1 |
|  |  |  |
| *8. Tremor* |  |  |
| Normal | 336/635 | 53 |
| Mild finger tremor, obvious to sight and touch | 228/635 | 36 |
| Tremor of hand or arm occurring spasmodically | 52/635 | 8 |
| Persistent tremor of one or more limbs | 17/635 | 3 |
| Whole body tremor | 2/635 | 0.3 |
|  |  |  |
| *9. Salivation* |  |  |
| Normal | 596/635 | 94 |
| Excess salivation so that drooling takes place if mouth is opened and tongue is raised | 34/635 | 5 |
| Excess salivation is present and might occasionally result in difficulty in speaking | 0/635 | 0 |
| Speaking with difficulty because of excess drooling | 0/635 | 0 |
| Frank drooling | 5/635 | 1 |
|  |  |  |
| MSAS original scoring median (IQR) | 0.20 | (0.10, 0.40) |
| MSAS omitting items 7 and 10 median (IQR) | 0.13 | (0.00, 0.38) |
|  |  |  |
| ***AIMS*** |  |  |
| *1. Muscles of Facial Expression* |  |  |
| None | 567/635 | 89 |
| Minimal | 34/635 | 5 |
| Mild | 24/635 | 4 |
| Moderate | 10/635 | 2 |
| Severe | 0/635 | 0 |
|  |  |  |
| *2. Lips and Perioral Area* |  |  |
| None | 415/635 | 65 |
| Minimal | 103/635 | 16 |
| Mild | 80/635 | 13 |
| Moderate | 32/635 | 5 |
| Severe | 5/635 | 1 |
|  |  |  |
| *3. Jaw* |  |  |
| None | 452/635 | 71 |
| Minimal | 94/635 | 15 |
| Mild | 58/635 | 9 |
| Moderate | 26/635 | 4 |
| Severe | 5/635 | 1 |
|  |  |  |
| *4. Tongue* |  |  |
| None | 419/635 | 66 |
| Minimal | 113/635 | 18 |
| Mild | 72/635 | 11 |
| Moderate | 28/635 | 4 |
| Severe | 3/635 | 0.5 |
|  |  |  |
| *5. Upper (arms, wrists, hands, fingers)* |  |  |
| None | 534/634 | 84 |
| Minimal | 58/634 | 9 |
| Mild | 35/634 | 6 |
| Moderate | 7/634 | 1 |
| Severe | 0/634 | 0 |
|  |  |  |
| *6. Lower (legs, knees, ankles, toes)* |  |  |
| None | 557/635 | 88 |
| Minimal | 44/635 | 7 |
| Mild | 27/635 | 4 |
| Moderate | 7/635 | 1 |
| Severe | 0/635 | 0 |
|  |  |  |
| *7. Neck, shoulders and hips* |  |  |
| None | 579/635 | 91 |
| Minimal | 24/635 | 4 |
| Mild | 20/635 | 3 |
| Moderate | 12/635 | 2 |
| Severe | 0/635 | 0 |
|  |  |  |
| *8. Severity of abnormal movements overall* |  |  |
| None | 371/635 | 58 |
| Minimal | 145/635 | 23 |
| Mild | 83/635 | 13 |
| Moderate | 31/635 | 5 |
| Severe | 5/635 | 1 |
|  |  |  |
| *9. Incapacitation due to abnormal movements* |  |  |
| None | 496/635 | 78 |
| Minimal | 84/635 | 13 |
| Mild | 36/635 | 6 |
| Moderate | 16/635 | 3 |
| Severe | 3/635 | 0.5 |
|  |  |  |
| *10. Patient's awareness of abnormal movements* |  |  |
| No awareness | 518/635 | 82 |
| Aware, no distress | 85/635 | 13 |
| Aware, mild distress | 25/635 | 4 |
| Aware moderate distress | 4/635 | 1 |
| Aware, severe distress | 3/635 | 0.5 |
|  |  |  |
| *11. Current problems with teeth and/or dentures* | 25/635 | 4 |
| Dentures usually worn | 9/635 | 1 |
| Endentia | 18/635 | 3 |
| Do movements disappear with sleep? – yes | 355/625 | 57 |
|  |  |  |
| AIMS score median (IQR) | 1 | (0, 6) |
| AIMS score using items 1 to 7 only median (IQR) | 0 | (0, 4) |
|  |  |  |
| ***BARS*** |  |  |
| *Objective* |  |  |
| Normal, occasional fidgety movements of the limbs | 444/635 | 70 |
| Presence of characteristic restless movements: shuffling or tramping movements of the legs/ feet, or swinging of one leg while sitting, *and/ or* rocking from foot to foot or “walking on the spot” when standing, but movements present for less than half the time observed | 130/635 | 20 |
| Observed phenomena, as described in (1) above, which are present for at least half the observation period | 45/635 | 7 |
| Patient is constantly engaged in characteristic restless movements, *and/ or* has the inability to remain seated or standing without walking or pacing, during the time observed | 16/635 | 3 |
|  |  |  |
| *Subjective* |  |  |
| Absence of inner restlessness | 387/635 | 61 |
| Non-specific sense of inner restlessness | 93/635 | 15 |
| The patient is aware of an inability to keep the legs still, or a desire to move the legs, *and/ or* complains of inner restlessness aggravated specifically by being required to stand still | 142/635 | 22 |
| Awareness of intense compulsion to move most of the time *and/ or* reports strong desire to walk or pace most of the time | 13/635 | 2 |
|  |  |  |
| *Distress related to restlessness* |  |  |
| No distress | 513/635 | 81 |
| Mild | 66/635 | 10 |
| Moderate | 36/635 | 6 |
| Severe | 20/635 | 3 |
|  |  |  |
| *BARS categorisation of akathisia* |  |  |
| Absent | 398/635 | 63 |
| Questionable | 80/635 | 13 |
| Mild | 108/635 | 17 |
| Moderate | 39/635 | 6 |
| Marked | 8/635 | 1 |
| Severe | 2/635 | 0.3 |
|  |  |  |
| *BARS akathisia positive (mild or greater)* | 157/635 | 25 |

**Supplementary Table 2**. Mixed effects linear regression model assessing the ability of the ScanMove instrument in predicting the score on the Modified Simpson Angus Scale, revised version with interactions (outcome measure). The model includes all 11 parkinsonism-specific ScanMove items.

|  | Revised scale, interactions | |
| --- | --- | --- |
| Variable | Coefficient | 95%CI |
| Item 1: Is the arm swing reduced (even on one side only)? | 0.10 | (0.05, 0.14) |
| Item 2: Is the stride length reduced (even on one side only)? | 0.03 | (-0.03, 0.10) |
| Item 3: Does the patient shuffle his/her feet? | 0.05 | (-0.06, 0.16) |
| Item 4: Does the patient walk with a stooped trunk? | -0.07 | (-0.14, 0.00) |
| Item 25: Do the patient’s finger tapping movements become smaller as he/she carries on with the task? | 0.02 | (-0.03, 0.08) |
| Item 26: If yes to item 25, does the patient’s finger tapping become also slower as he/she carries on with the task? | 0.01 | (-0.05, 0.08) |
| Item 27: Do the patient’s foot tapping movements become smaller as he/she carries on with the task? | 0.02 | (-0.05, 0.10) |
| Item 28: If yes to item 27, does the patient’s foot tapping become also slower as he/she carries on with the task? | -0.03 | (-0.10, 0.04) |
| Item 31: Do you notice any excessive pooling of saliva in the mouth, or is there any drooling of saliva outside of his/her mouth? | -0.17 | (-0.32, -0.02) |
| Item 32: Is his/her voice excessively soft? | -0.12 | (-0.23, -0.00) |
| Item 33: With the patient relaxed and not actively contracting his/her muscles, do you feel any resistance while doing these manoeuvres? | 0.08 | (0.01, 0.14) |
|  |  |  |
| Interactions |  |  |
| Item_2 item_4 | 0.22 | (0.10, 0.34) |
| Item _3 item_4 | 0.16 | (0.02, 0.29) |
| Item_3 item_25 | -0.13 | (-0.26, -0.01) |
| Item _3 item_31 | 0.44 | (0.15, 0.72) |
| Item _4 item_31 | 0.25 | (0.01, 0.50) |
| Item _26 item_27 | 0.12 | (0.02, 0.21) |
| Item _26 item_33 | 0.14 | (0.04, 0.23) |
| Item _27 item_31 | 0.56 | (0.19, 0.93) |
| Item _28 item_31 | -0.43 | (-0.85, -0.02) |
| Item _28 item_32 | 0.28 | (0.08, 0.47) |

**Supplementary** **Table 3.** Mixed effects linear regression model assessing the ability of the ScanMove instrument in predicting the score on the Abnormal Involuntary Movements Scale, revised version with interactions (outcome measure). The model includes all 14 dyskinesias-specific ScanMove items.

|  | Revised scale, interactions | |
| --- | --- | --- |
| Variable | Coefficient | 95%CI |
| Item 5: *While walking* Is the patient’s head tilting back or to one side? | -0.54 | (-1.65, 0.56) |
| Item 6: *While walking* Do you notice any abnormal movements of the face (such as grimacing, pursing and smacking of the lips, chewing and lateral movements of the jaw, tongue protrusion)? | 1.71 | (0.74, 2.68) |
| Item 7: *While walking* Do you notice any abnormal movements of the limbs (such as shaking, twitching or twisting of hands or feet)? | -0.99 | (-1.74, -0.23) |
| Item 13: *While standing* Is the patient’s head tilting back or to one side? | 0.73 | (-0.64, 2.10) |
| Item 14: *While standing* Do you notice any abnormal movements of the face (such as grimacing, pursing and smacking of the lips, chewing and lateral movements of the jaw, tongue protrusion)? | 2.48 | (1.25, 3.70) |
| Item 15: *While standing* Do you notice any abnormal movements of the limbs (such as shaking, twitching or twisting of hands or feet)? | 0.61 | (-0.07, 1.29) |
| Item 20: *While sitting* Is the patient’s head tilting back or to one side? | 0.47 | (-0.92, 1.86) |
| Item 21: *While sitting* Do you notice any abnormal movements of the face (such as grimacing, pursing and smacking of the lips, chewing and lateral movements of the jaw, tongue protrusion)? | 0.01 | (-1.00, 1.03) |
| Item 22: *While sitting* Do you notice any abnormal movements of the limbs (such as shaking, twitching or twisting of hands or feet)? | 0.19 | (-0.54, 0.93) |
| Item 29: *While keeping mouth open* Do you notice any abnormal movements of the face (such as grimacing, pursing and smacking of the lips, chewing and lateral movements of the jaw, tongue protrusion)? | 0.26 | (-0.60, 1.11) |
| Item 34: *While holding arms outstretched or in front of chest with each elbow out to the side* Is the patient’s head tilting back or to one side? | -0.30 | (-1.90, 1.31) |
| Item 35: *While holding arms outstretched or in front of chest with each elbow out to the side* Do you notice any abnormal movements of the face (such as grimacing, pursing and smacking of the lips, chewing and lateral movements of the jaw, tongue protrusion)? | 0.96 | (-0.04, 1.96) |
| Item 36: *While holding arms outstretched or in front of chest with each elbow out to the side* Do you notice any abnormal movements of the limbs (such as shaking, twitching or twisting of hands or feet)? | -0.11 | (-0.65, 0.43) |
| Item 38: Do you notice any abnormal shaking, twitching, or twisting of the hands while writing or drawing? | -0.05 | (-0.72, 0.63) |
|  |  |  |
| Interactions |  |  |
| Item 7 item 20 | 4.13 | (1.80, 6.46) |
| Item 13 item 22 | -5.67 | (-8.36, -2.97) |
| Item 13 item 35 | 4.63 | (2.24, 7.02) |
| Item 14 item 34 | -8.03 | (-11.73, -4.32) |
| Item 14 item 35 | -2.93 | (-4.46, -1.40) |
| Item 15 item 21 | 2.13 | (0.96, 3.30) |
| Item 20 item 21 | -3.20 | (-5.31, -1.09) |
| Item 22 item 34 | 3.39 | (0.84, 5.95) |
| Item 22 item 38 | 1.19 | (0.14, 2.24) |
| Item 29 item 35 | 2.57 | (1.23, 3.91) |

**Supplementary Table 4.** Mixed effects linear regression model assessing the ability of the ScanMove instrument in predicting the score on the Barned Akathisia Rating Scale (dichotomous outcome measure). The model includes all 6 akathisia-specific ScanMove items.

|  | Accounting for clustering | |
| --- | --- | --- |
| Variable | OR | 95%CI |
| Item 9: *While standing* Does the patient have any purposeless movements of the legs, such as marching or stamping movements, walking on-the-spot, twitchy, jerky movements? | 1.98 | (1.04, 3.78) |
| Item 10: *While standing* Does the patient’s body keep rocking side to side? | 3.60 | (1.64, 7.92) |
| Item 11: *While standing* Does the patient keep pacing around the room leaving his/her spot despite the instruction to stand still? | 0.17 | (0.03, 1.16) |
| Item 17: *While sitting* Does the patient have any purposeless movements of the legs, such as shuffling, jiggling, trampling of the legs? | 7.00 | (3.37, 14.53) |
| Item 18: *While sitting* Does the patient get up out of the chair despite the instruction to sit down? | 13.97 | (0.92, 211.26) |
| Item 24: *While sitting* Does the patient’s body keep rocking side to side? | 1.03 | (0.26, 4.15) |
|  |  |  |
| Area under curve | 0.72 | (0.67, 0.77) |
| Optimum sensitivity (%)* | 63.82 | (55.64, 71.44) |
| Optimum specificity (%)* | 67.82 | (63.35, 72.06) |
| Correctly classified (%)* | 66.83 | (62.95, 70.54) |

*Optimum sensitivities and specificities were at the point where sensitivity+specificity was maximised.
